# Coproantigen detection and molecular identification of *Cryptosporidium* species among newborn and adult farm animals

**DOI:** 10.1186/s13568-024-01817-x

**Published:** 2025-01-22

**Authors:** Dina Aboelsoued, Nagwa I. Toaleb, Kadria N. Abdel Megeed

**Affiliations:** Parasitology and Animal Diseases Department, Veterinary Research Institute, National Research Centre, El Buhouth St., Dokki, Giza Egypt

**Keywords:** *Cryptosporidium*, Sandwich ELISA, PAbs, PCR, *C. parvum*, *C. hominis*, *C. andersoni*

## Abstract

*Cryptosporidium* sp. is an obligatory intracellular apicomplexan protozoan parasite that causes a disease called cryptosporidiosis with substantial veterinary and medical importance. Therefore, this study aimed to evaluate an early diagnosis of cryptosporidiosis using the anti-*Cryptosporidium parvum* oocyst immunoglobulin IgG polyclonal antibodies (anti-*C. parvum* IgG PAbs)-based sandwich enzyme-linked immunosorbent assay (ELISA) for the detection of *Cryptosporidium* oocyst antigens in fecal samples of farm animals in Egypt. Further molecular identification and sequencing were performed for the detected isolates. Eight hundred and twenty fecal samples of farm animals; 102 buffalo calves, 120 cattle calves, 100 lambs and 98 goat kids, 80 buffaloes, 60 cattle, 160 sheep and 100 goats, collected from different small-scale farms and local holders were examined for cryptosporidiosis by Modified Ziehl-Neelsen (MZN) technique. The percentage of positivity was 45.1%, 50%, 20%, 18.4%, 31.25%, 38.3%, 18.8%, and 11% in buffalo calves, cattle calves, lambs, goat kids, adult buffaloes, adult cattle, sheep, and goats, respectively. Molecular identification of *Cryptosporidium* samples was performed based on COWP gene, revealing the isolates: GenBank: OQ121955.1, OR029973.1 and PP316107.1 which were identical to the *C. parvum* and GenBank: PP316108.1 and OR029972.1 which were identical to *C. hominis* and *C. andersoni*, respectively. Then, *C. parvum* oocysts were used for preparation of antigens and rabbit immunization. Anti-*C. parvum* IgG PAbs were purified and characterized by SDS-PAGE and then labeled with horseradish peroxidase (HRP). Anti-*C. parvum* IgG PAbs in-house sandwich ELISA was prepared, then tested this ELISA on 820 samples and compared results with MZN microscopical examination and a commercial sandwich ELISA kit. In this study, in-house sandwich ELISA scored higher sensitivity of 98%, 100% specificity, validity 99% and relative agreement 98.6% than (92%, 90%, 91% and 91.4%) of MZN and (96%, 95%, 95.5% and 95.7%) of coproantigen commercial sandwich ELISA kit, respectively. Moreover, we used PCR to evaluate the positivity of in-house sandwich ELISA results, and the total PCR positive samples were 263 out of 268 sandwich ELISA positive samples (98.13%). **In conclusion**, the prepared Anti-*C. parvum* IgG PAbs based sandwich ELISA offered a simple and accurate diagnostic method for cryptosporidiosis in the fecal samples of different species of farm animals in Egypt with high sensitivity (98%) and specificity (100%). Further studies on this Anti-*C. parvum* IgG PAbs may help also in the protection against cryptosporidiosis.

## Introduction

*Cryptosporidium* spp. are intestinal zoonotic protozoans responsible for gastroenteritis in a broad range of animals and humans (Santín 2020). They are the leading cause of morbidity and mortality in children under 5 years of age (Khalil et al. 2018) and cattle neonatal enteritis (Thomson et al. 2017). The pathogenicity of cryptosporidiosis varies with species *Cryptosporidium* species, age and immune status of the host (Feng et al. 2018). Its transmission happens by fecal oral route, or by oocyst-contaminated food and water (Feng et al. 2018). *Cryptosporidium* is a main cause of waterborne outbreaks in industrialized nations (Zahedi and Ryan 2020). Its economic impact is attributed to high morbidity and mortality, low performance, increased labor needed and the high cost of treatment (Wegayehu et al. 2013) in addition to threatening public health by spreading through water sources (Chen et al. 2022).

Diagnosis of protozoan parasites could be performed by parasitological methods or using serum circulatory antigens and/or antibody detection (Farid 2023). For cryptosporidiosis, microscopical examination of stained slides from fecal samples, mostly using modified Ziehl-Neelsen (MZN) staining technique, with a sensitivity of about 75% (Chalmers et al. 2011) is the most used diagnostic method. Unfortunately, this method of diagnosis requires experts to detect the presence of *Cryptosporidium* and the phase of its life cycle (McHardy et al. 2014; Elmahallawy et al. 2022) as well as it needs at least 5 × 10^4^ oocysts per mL of feces to be detected. Due to the tiny size of *Cryptosporidium* oocysts, the differential staining by MZN or wet mount preparation methods can be easily misdiagnosed with other materials present in fecal samples (Connelly et al. 2008). Also, since the oocysts don’t shed regularly, at least three samples collected in different days could be optimum (Farid et al. 2023).

To get around all these disadvantages of microscopical examination, antigen/antibody-based detection methods could be used (Vanathy et al. 2017). Immunodiagnostic techniques offer higher sensitivity and early identification of *Cryptosporidium*(Aboelsoued et al. 2022a). These techniques could detect antibodies or antigens using enzyme-linked immunosorbent assay (ELISA), western blots, and/or immunofluorescence assays (IFA) (Júlio et al. 2012). Antibody screening tests are successful detectors in severe infections, but they could have cross-reactivity with other protozoans (Pacheco et al. 2020) so, they might run the risk of returning a false positive result. Sandwich ELISA approach with polyclonal antibodies (PAbs) succeeded in detecting many parasite types (Maher et al. 2022; Toaleb et al. 2023; Maher et al. 2024). In addition, antigen screening techniques are recommended because they could detect acute infection as well as having high sensitivity Khurana and Chaudhary (2018).

Polymerase Chain Reaction (PCR) techniques have also the advantage of improved sensitivity (97–100%) and specificity (100%). PCR was the best techniques to monitor *Cryptosporidium* oocysts in feces of various animal hosts such as: sheep, horses, buffaloes and cattle (Mirhashemi et al. 2015; Abu El Ezz et al. 2018; Elmahallawy et al. 2022). PCR tests could diagnose mixed infections and offer a better knowledge about livestock species diversity (Ahmed and Karanis 2018). However, they have limited availability at poor settings due to their costs, high technical expertise involved and infrastructure needs (Omoruyi et al. 2014), also, sometimes it’s hard in calves with inapparent or subclinical infection (Wang et al. 2021).

Exploring an effective and early diagnosis might be a key step to prevent cryptosporidiosis. Therefore, this work aimed to evaluate purified anti*-Cryptosporidium* oocyst IgG PAbs as an early diagnostic marker of cryptosporidiosis. An in-house sandwich ELISA based on IgG PAbs was developed to detect *Cryptosporidium* oocyst antigens in farm animals’ (buffalo calves, cattle calves, lambs, goat kids, adult cattle, adult buffaloes, sheep and goats) fecal samples. In addition, PCR was utilized to identify the *Cryptosporidium* predominant species infecting farm animals.

## Materials and methods

### Animals

A total number of 820 farm animals; 102 buffalo calves (aged from 1 day to 6 months), 120 cattle calves (from 1 day to 6 months), 100 lambs (less than 12 month), 98 goat kids (up to 9 weeks), 80 buffaloes, 60 cattle, 160 sheep, 100 goats, from different small-scale farms and local holders in: Cairo (30°02′N, 31°13′E), Giza (29° 58’ 27.00” N, 31° 08’ 2.21” E) and Beni-Suef (29° 03’ 60.00” N, 31° 04’ 60.00” E) governorates, Egypt, were included in this study during the period from April 2022 to December 2023.

### Fecal samples

For this study, fresh fecal samples were collected directly from the rectum of each animal with permission from the animal owners for collecting fecal samples. Each sample was kept in separate clean labeled containers and transferred in ice boxes to the Immunology and Parasitology Laboratory, National Research Centre (NRC), Egypt. The collected samples were prepared and examined on the day of collection.

### Parasitological examination

Animal fecal smears were smeared on glass slides, prepared, stained by MZN (Henriksen and Pohlenz 1981) then examined under light microscope (oil immersion). Infection severity was determined by counting the *Cryptosporidium* oocysts in a field at 1000× magnification; the scores were mild when 2–6 oocysts/field, moderate when 7–12 oocysts/field, and severe when more than 12 oocysts/field were detected (Anderson 1981).

### Isolation of *Cryptosporidium* sp. oocysts

*Cryptosporidium* oocysts were concentrated from severe *Cryptosporidium-*positive feces by flotation using Sheather’s sugar solution (Current and Reese 1986), collected, and then stored in Potassium dichromate solution (2.5%; Sigma-Aldrich, Canada) at 4 °C.

### Genomic DNA extraction

Using a DNA extraction kit (GeneDireX, USA), DNA was extracted from 50 isolated oocyst samples from buffalo calves, cattle calves, adult buffaloes, sheep and goats (10 samples each) for identification of *Cryptosporidium* spp. Also, DNA was extracted from fecal samples of (*n* = 268: 50 buffalo calves, 69 cattle calves, 23 lambs, 25 goat kids, 28 buffaloes, 26 cattle, 33 sheep and 14 goats) to evaluate the positivity of the sandwich ELISA results by PCR. After extraction, the DNA concentration was estimated by a spectrophotometer (microvolume Q9000, Quawell, USA) and then stored at − 20 °C.

### PCR

Identification of the extracted DNA was done using PCR targeting the *Cryptosporidium* oocyst wall protein (COWP) gene according to Feltus et al. (2006) and (Aboelsoued et al. 2022b) in a thermal Cycler (BIO-RAD, Singapore). Then, PCR products were visualized by a Molecular Imager (BIO RAD, Singapore) in agarose gel electrophoresis (1.5%) stained with RedSafe (Intron Biotechnology, Republic of Korea), with a 100 bp ladder (QIAGEN, USA).

### Sequencing

PCR products were purified using gel extraction kit (GeneDireX, USA) according to manufacturer’s protocol. The purified products were sequenced by sequencing kit (Big Dye Terminator V3.1 Cycle, Perkin-Elmer, USA) with ABI 3130 automated sequencer (Applied Biosystems, USA). Then, sequences were corrected using ChromasPro 1.7 software (Technelysium Pty Ltd., Australia) and compared to those available in GenBank with nucleotide BLAST (https://blast.ncbi.nlm.nih.gov/Blast.cgi*)* and submitted in GenBank. Multiple sequences alignment was performed using CLUSTAL W 1.83 of MegAlign module of Lasergene DNAStar software Pairwise following Thompson et al. (1994). Phylogenetic analysis was done with maximum likelihood, neighbor-joining and maximum parsimony according to Tamura et al. (2013) in MEGA6 software.

### Mice experimental infection

Fifty parasite-free mice (Swiss albino) were experimentally infected with a dose of 10^4^*C. parvum* oocysts isolated from cattle calves by gastric tubes in a single dose one hour before meal (Aboelsoued et al. 2023). Animal fecal pellets were collected every day starting from the third day of infection for three weeks and examined by MZN staining (Henriksen and Pohlenz 1981).

### *Cryptosporidium* oocyst antigen preparation

The isolated *C. parvum* oocysts from mice fecal samples were washed three times with Phosphate buffered saline (PBS; 16000 rpm/20 min), then resuspended in PBS and freeze-thawed for 20 cycles. The oocysts were then sonicated for 15 cycles/30 seconds each and centrifuged (16000 rpm/20 min at 4 °C). Then, the collected supernatant (*C. parvum* oocyst antigen) was frozen at − 20 °C (Kaushik et al. 2009; Aboelsoued et al. 2022a) after protein concentration determination as described by Lowry’s method (Lowry et al. 1951).

### Preparation of anti-*C. parvum* oocyst antigen IgG PAbs

The anti-*C. parvum* oocyst antigen IgG polyclonal antibodies (anti-*C. parvum* IgG PAbs) were produced in five healthy male rabbits (New Zealand) free of parasitic infections (about 2 months of age and weighing 1.5–2.0 Kg) according to Fagbemi et al. (1995). Blood samples were collected from rabbit’s ear vein four days after the last injection and sera were separated from blood. Sera were tested by indirect ELISA with the corresponding *C. parvum* oocyst antigen checking for the presence of antibodies, then aliquoted and stored frozen at − 20 °C until use.

### Purification of anti-*C. parvum* IgG PAbs using protein-A affinity chromatography

Anti-*C. parvum* antigen IgG PAbs was purified from the prepared rabbit hyperimmune sera according to Toaleb et al. (2023) and Farid et al. (2023). In brief, anti-*C. parvum* IgG PAbs was precipitated in rabbit hyperimmune serum by adding 50% saturated Ammonium sulphate solution in which most albumin was removed from anti-*C. parvum* IgG. The anti-*C. parvum* IgG PAbs was then dialyzed using 0.15 M PBS, pH = 7.2 for 3 days at 4 °C to remove Ammonium sulfate salt. After that, the supernatant was concentrated using polyethylene glycol (Abd El Hafez et al. 2010). For more purification, protein-A Sepharose gel affinity chromatography was used to purify anti-*C. parvum* antigen IgG PAbs as described by Abd El Hafez et al. (2010). The protein concentration of the purified anti-*C. parvum* IgG PAbs was estimated following Lowry et al. (1951).

### SDS-PAGE

Electrophoresis of purified anti-*C. parvum* IgG PAbs was performed in 10% polyacrylamide gels under reducing conditions as described by Laemmli (1970). After separation, slab gel was stained with Coomassie Brilliant Blue dye. Molecular weights were analyzed using Gel Doc™ XR + Imager (Bio-Rad, California, USA). Relative bands’ molecular weights were estimated using prestained protein marker (GeneDirex BLUltra, USA) in the same gel.

### Conjugation of anti-*C. parvum* IgG PAbs with Horseradish Peroxidase (HRP) Enzyme

Labeling of the anti-*C. parvum* IgG PAbs with HRP enzyme was performed as described by Toaleb et al. (2023). Briefly, about 10 mg of HRP enzymes were mixed with 5 mg of anti-*C. parvum* IgG PAbs in 1 mL of 0.1 M PBS (pH = 6.8). The mixture was dialyzed overnight at 4 °C. Diluted glutaraldehyde (Sigma-Aldrich) was added to the dialyzed mixture with gentle stirring for 3 h at room temperature (Avrameas 1969). Then 2 M glycine solution was added to the mixture and left at room temperature for 2 h then dialyzed overnight at 4 °C against 0.1 M PBS (pH = 6.8). The mixture was centrifuged for 30 min/16,000 rpm at 4 °C. The prepared conjugated anti-*C. parvum* IgG PAbs HRP was aliquoted and stored at ‒20 °C until use.

### Indirect ELISA

Indirect ELISA was used to evaluate the reactivity of the produced anti-*C. parvum* IgG PAbs in rabbit hyperimmune serum according to Engvall and Perlman (1971). Dilutions of antigen, hyperimmune sera and anti-rabbit conjugate (Sigma-Aldrich) were chosen based on checkerboard titration. The optical density (OD) values were measured at 450 nm by a microplate reader (ELx808UV, BioTek Instruments, USA) using Ortho-phenylenediamine (OPD; Sigma-Aldrich) substrate to develop the color of the reaction.

### In-house sandwich ELISA

Coproantigen sandwich ELISA was performed to detect *C. parvum* antigen in the collected fecal samples as described by Duménigo et al. (1996) with some modifications. Briefly, the purified anti-*C. parvum* IgG PAbs was applied to the ELISA microtiter plates (100 µl/well in Carbonate buffer) and incubated at 4^○^C overnight. Then the plates were washed twice/5 min each with washing buffer. 200 µl of blocking buffer (containing 3% skimmed milk, Sigma Aldrich) was added to block the nonspecific reactive sites and incubated for 2 h at room temperature. After washing three times/5 min, 100 µl of the diluted fecal supernatant sample (1:10 in PBS-Tween) was added in triplicates and the plates were incubated for 1.5 h at 37^○^C. After incubation, solutions were removed, and the plates were washed 3 times with PBS-Tween. Then, 100 µl/well of diluted anti-*C. parvum* IgG HRP conjugate was dispensed, and the plates were incubated at 37^○^C for 1 h. Plates were washed three times again with PBS-Tween. The reaction was visualized by adding 100 µl/well of OPD (Sigma-Aldrich) 1 mg/mL in substrate solution for 20–30 min in the dark at room temperature. Optimal concentrations of specific purified anti-*C. parvum* IgG PAbs, fecal samples dilutions, and anti-*C. parvum* IgG PAbs HRP conjugate were determined by checkerboard titration. The reaction was stopped by 50 µL/well of 2 M Sulfuric acid. OD values were measured at 450 nm using ELISA reader (ELx808 UV, BioTek Instruments, USA). The cut off value was measured as the mean OD of the negative control samples plus three times the standard deviation (3 SD) (Aboelsoued et al. 2024).

Using in-house sandwich ELISA, the specificity and cross reactivity of the purified anti‑*C. parvum* IgG PAbs in the detection of the *Cryptosporidium* oocysts antigen was validated using positive *C. parvum*-infected cattle calves’ fecal samples (OR029973.1; *n* = 25), and other parasite antigens in fecal samples [*Giardia intestinalis* infected sheep fecal samples (OQ388332.1; *n* = 15), *Entamoeba histolytica* sheep infected fecal samples (OR016432.1; *n* = 13), *Fasciola gigantica* in cattle fecal samples (*n* = 12)], compared to non-infected fecal samples [cattle and sheep fecal samples 10/each (*n* = 20)]. The ELISA plate was coated with purified anti‑*C. parvum* IgG PAbs and the prepared anti-*C. parvum* IgG PAbs HRP was used as a conjugate.

Sensitivity, specificity, diagnostic efficacy, and accuracy percentages of MZN, in-house sandwich ELISA and commercial sandwich ELISA kit in the detection of *Cryptosporidium* sp. were estimated using 50 experimentally infected mice fecal samples collected at the10th day post infection (PI; peak of oocyst shedding) according to Schechter (1998).

### Commercial sandwich ELISA kit

A commercial sandwich ELISA kit (INOVA BIOTECH CO. Ltd., Beijing, China) was used to detect *C. parvum* in fecal samples following the manufacturer’s instructions. The plates are previously coated with an antibody specific to *Cryptosporidium* (CPS). In our lab, fecal samples were diluted and added to each well. The HRP conjugated *Cryptosporidium* antibody appeared blue in color and then turned yellow after adding stop solution. OD was measured using Microplate ELISA reader at a wavelength of 450 nm. The presence of *Cryptosporidium* was determined by comparison with the cutoff value (the average value of negative control + 0.15).

### Statistical analysis

Statistical analyses were performed using SPSS Software V.19 for Windows (IBM, NY, USA). Results of conventional MZN, in-house sandwich ELISA and the Commercial ELISA kit were analyzed using Chi-square test.

## Results

### Detection of oocysts using MZN

**Fig. 1:**
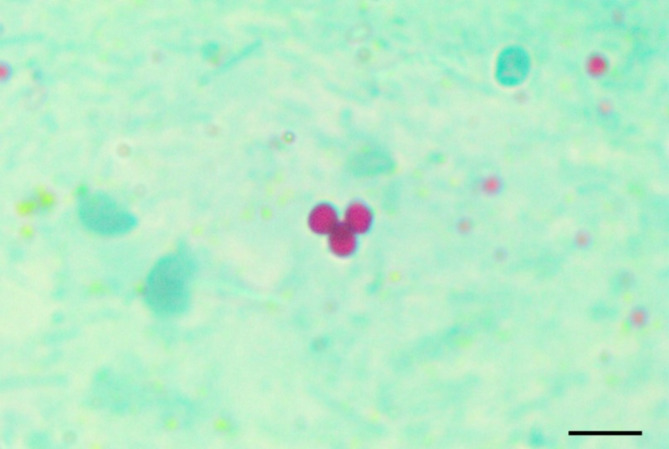
*Cryptosporidium* spp. oocysts in fecal smear, stained by modified Ziehl–Neelsen techniques, stained pink against green fecal debris and yeasts (×1000; Scale Bar = 0.4 μm)

*Cryptosporidium* sp. oocysts containing 4 sporozoites appeared as pink spherical bodies 4–6 μm against a green background (Fig. 1). Examination of 820 fecal samples for *Cryptosporidium* oocysts revealed that 233 fecal samples were found to be infected with *Cryptosporidium* spp. (28.4%) with an overall infection rate: 45.1% (46/102) in buffaloes calves, 50% (60/120) in cattle calves, 20% (20/100) in lambs, 18.4% (18/98) in goat kids, 31.25% (25/80) in adult buffaloes, 38.3% (23/60) in adult cattle, 18.8% (30/160) in sheep, and 11% (11/100) in goats.

### Molecular identification of the isolated *Cryptosporidium* spp

**Fig. 2:**
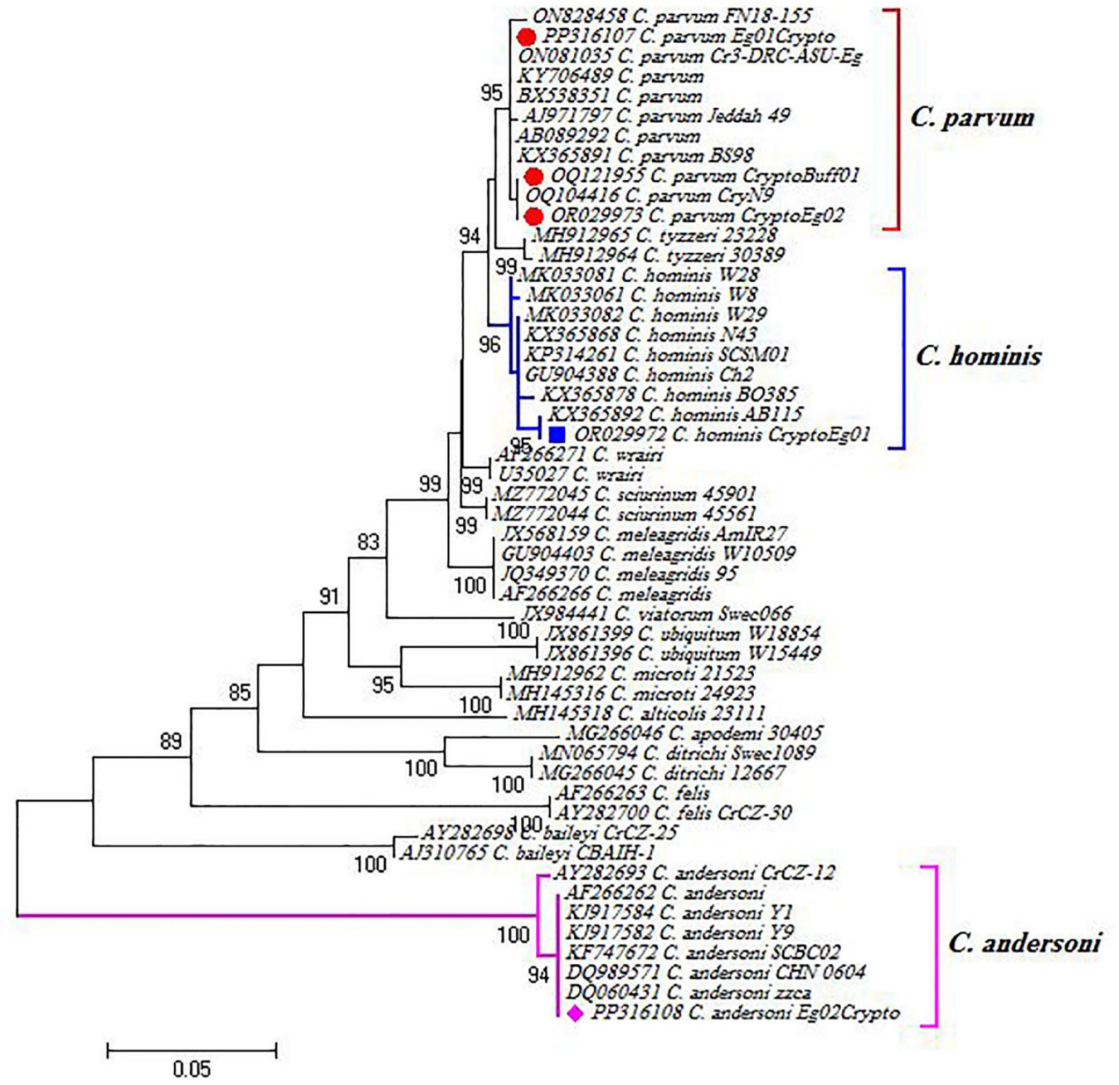
Phylogenetic analysis using the maximum likelihood method based on COWP gene for *Cryptosporidium* sp. Our obtained isolates, clustered in well-supported branches with other *Cryptosporidium* references, are highlighted (red circle: *C. parvum*, blue square: *C. hominis* and pink diamond: *C. andersoni*). The scale bar represents a 5% nucleotide sequence divergence

*Cryptosporidium* DNA was detected in the fecal samples of heavily infected buffalo calves, cattle calves, adult buffaloes, sheep and goat (10 animals each) using the COWP gene. The BLAST search revealed five genotypes, and their sequences were deposited in GenBank. These genotypes were *C. parvum* in the buffalo calves (GenBank: OQ121955.1), cattle calves (GenBank: OR029973.1) and adult buffaloes (GenBank: PP316107.1). Also, we detected *C. andersoni* in goats (GenBank: PP316108.1) and *C. hominis* in sheep (GenBank: OR029972.1). Phylogenetic analysis revealed that these genotypes clustered in a well-supported branch with other *Cryptosporidium* sp. sequences (Fig. 2).

### Protein content

Protein content was 5.4 mg/ml in crude *Cryptosporidium* oocysts antigen, 8.6 mg/ml in crude anti*-Cryptosporidium* oocysts PAbs in rabbit hyperimmune serum and 2.4 mg/ml after purification procedure.

### Immuno-reactivity of anti‑*C. parvum* oocysts IgG PAbs

**Fig. 3:**
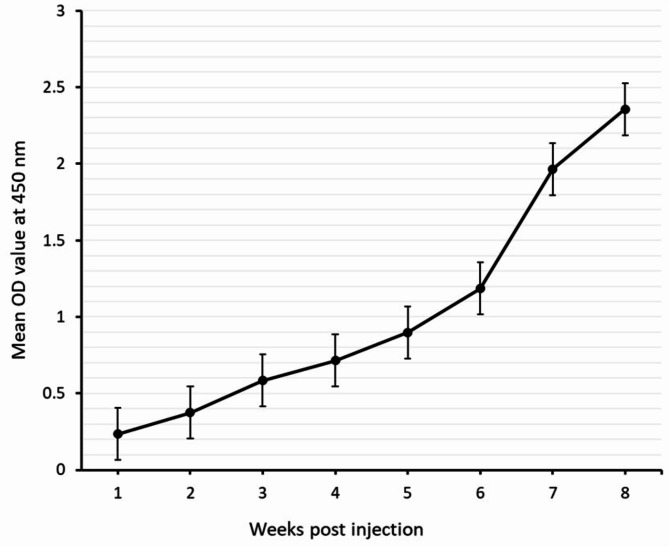
Reactive binding activities of prepared anti‑*C. parvum* oocysts IgG PAbs in rabbit hyperimmune sera against *C. parvum* oocyst antigen measured by indirect ELISA

Indirect ELISA was adopted to check the immunoreactivity and level of the produced anti‑*C. parvum* oocysts’ antigen IgG PAbs against *C. parvum* oocysts’ antigen in rabbits. This level elevated starting one week of the 1st booster dose and continued until reached OD value = 2.356 (each OD value represented the mean OD value of five immunized rabbit sera) as shown in Fig. 3.

### Characterization of the purified anti‑*C. parvum* oocysts IgG PAbs

**Fig. 4:**
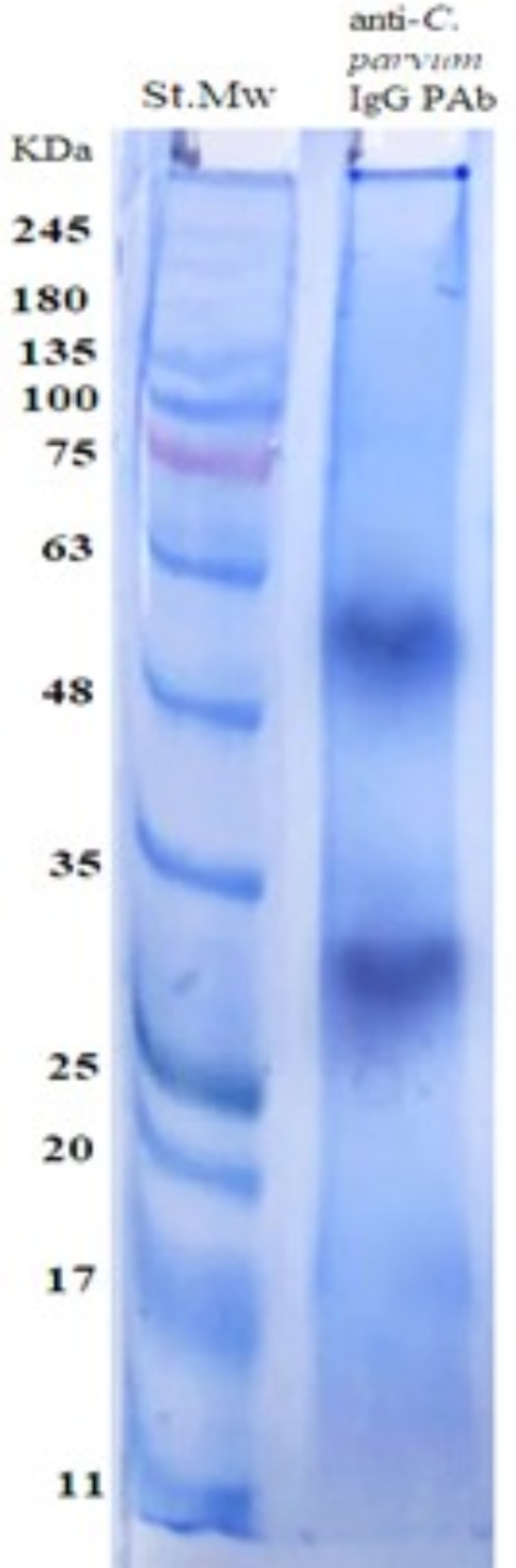
Electrophoretic profile of anti-*C. parvum* IgG PAbs (Lane: anti-*C. parvum* IgG PAbs) and Molecular weight protein standard (Lane: St. Mr)

The electrophoretic profile of the purified anti‑*C. parvum* oocysts IgG PAbs by SDS-PAGE showed that the pure anti‑*C. parvum* IgG PAbs was free from any other proteins and was represented by two bands: the heavy chain 50 kDa and the light chain 30 KDa of immunoglobulin (Fig. 4).

### Diagnostic potentials and binding activities of purified anti‑*C. parvum* IgG PAbs

**Fig. 5:**
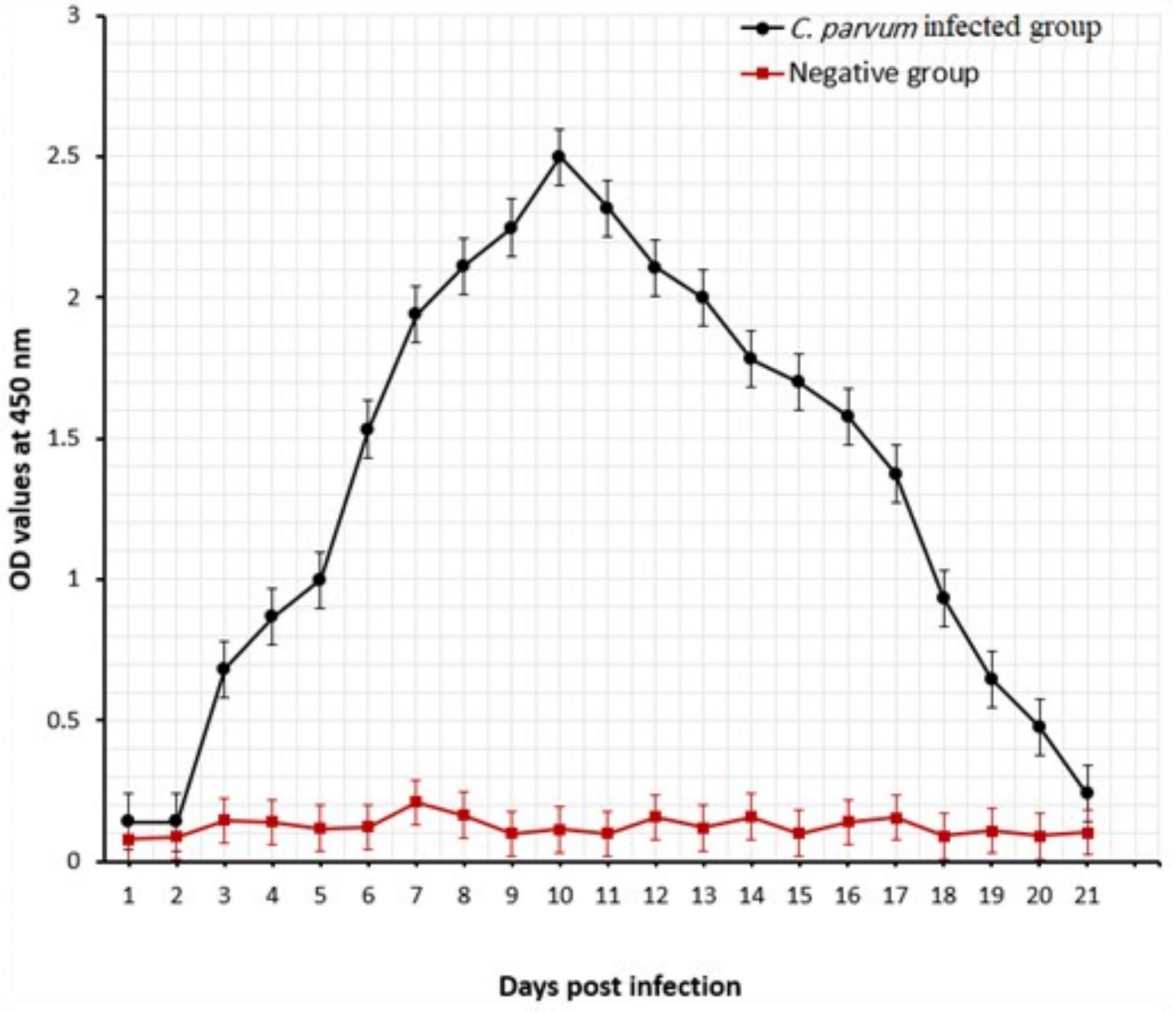
Potency of the purified anti‑*C. parvum* IgG PAbs to detect *C. parvum* oocysts antigen in experimentally infected mice fecal samples (●) and healthy mice fecal samples (■) by sandwich ELISA, OD values represent mean OD value ± SD

The diagnostic success of the purified anti‑*C. parvum* IgG PAbs to detect *C. parvum* antigen in the fifty experimentally infected mice feces and healthy mice fecal samples was proved by Sandwich ELISA. The binding activity of anti‑*C. parvum* IgG PAbs with *C. parvum* oocysts’ antigen increased gradually starting from the third day of infection till reached its maximum OD values at the10th day PI (peak of oocyst shedding; OD = 2.497) as shown in Fig. 5.

### Specificity of purified anti‑*C. parvum* IgG PAbs in the diagnosis of cryptosporidiosis

**Fig. 6:**
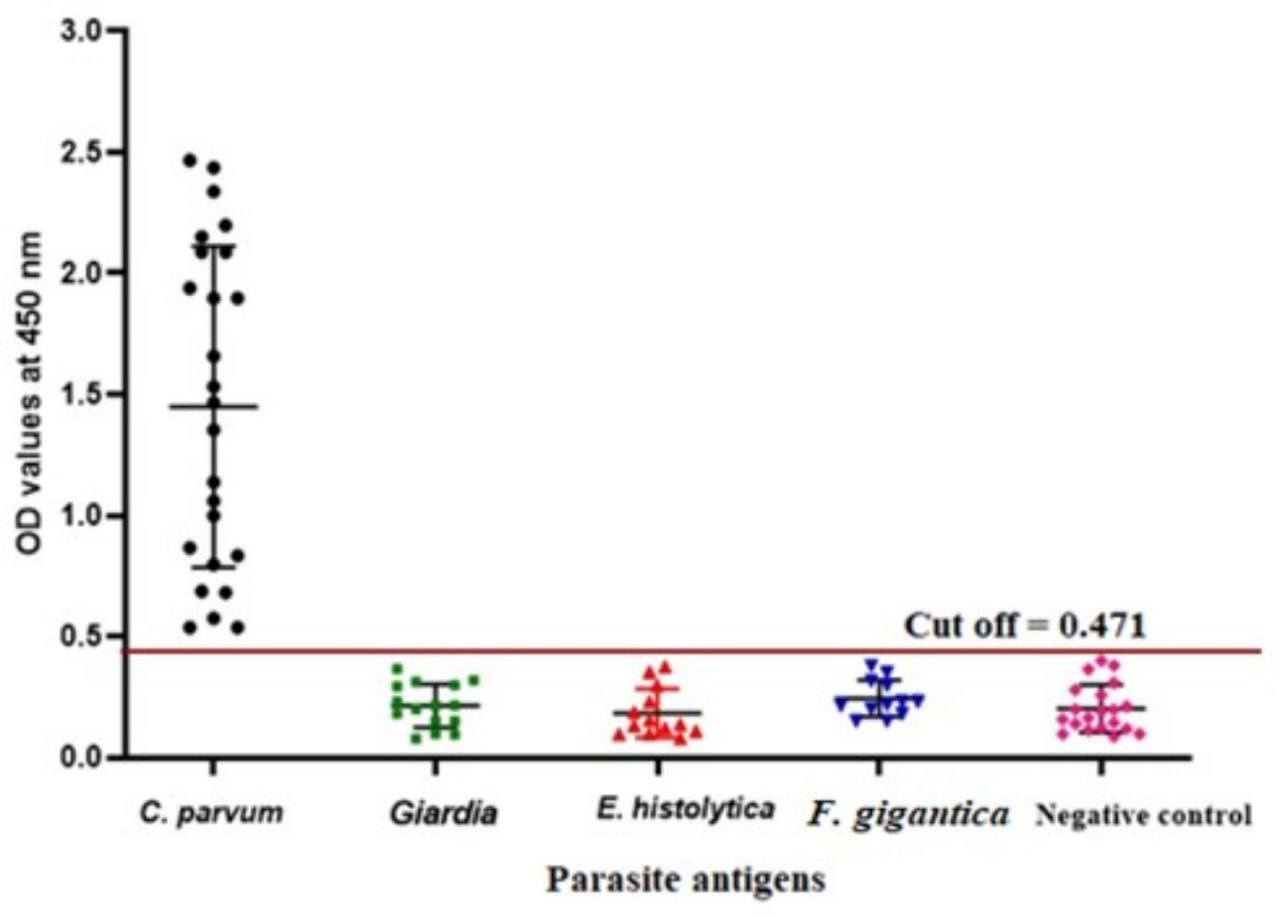
Comparative evaluation of the specificity of purified anti‑*C. parvum* IgG PAbs in detecting *Cryptosporidium* oocysts’ antigen and other parasite antigens

The anti‑*C. parvum* IgG PAbs showed no cross reaction with the other parasites’ antigens as well as the negative control, while it revealed a strong reactivity towards *Cryptosporidium* oocyst antigen samples. The OD readings at 450 nm for *Cryptosporidium* infected samples ranged from 0.575 to 2.466 (mean OD value = 1.689 ± 0.618). For other parasite groups and healthy control samples were 0.213 ± 0.086, 0.183 ± 0.096, 0.228 ± 0.086, and 0.213 ± 0.085 for *G. intestinalis*, *E. histolytica*, *F. gigantica*, and healthy control, respectively, which were lower than the cut off value (0.471) (Fig. 6).

Assessment of MZN, in-house sandwich ELISA and commercial sandwich ELISA kit in diagnosis of *Cryptosporidium* sp

The sensitivity of MZN, in-house sandwich ELISA and commercial sandwich ELISA kit in the detection of *Cryptosporidium* sp. in 50 experimentally infected mice fecal samples was 92%, 98%, and 96%, respectively. In contrast, their specificity was 90%, 100%, and 95%, respectively. Positive predictive values (PPV) were 95.8%, 100%, and 97.9% while negative predictive values (NPV) were 81.8%, 95.2%, and 90.5% and their diagnostic efficacy was 91.4%, 98.6% and 95.7% for MZN, in-house sandwich ELISA and commercial sandwich ELISA kit, respectively (Table 1).

**Table 1:** Comparative performance of MZN, in-house sandwich ELISA and commercial sandwich ELISA in the detection of *Cryptosporidium* antigen in *C. parvum-*experimentally infected mice fecal samples

Diagnostic values	Diagnostic test		
	MZN	In-house sandwich ELISA	Commercial sandwich ELISA kit
Sensitivity	92%	98%	96%
Specificity	90%	100%	95%
Positive predictive value % (PPV)	95.8%	100%	97.9%
Negative predictive value % (NPV)	81.8%	95.2%	90.5%
Diagnostic efficacy	91.4%	98.6%	95.7%
Validation	91%	99%	95.5%
Relative agreement	91.4%	98.6%	95.7%

### Antigen-based diagnosis of cryptosporidiosis in farm animals’ fecal samples

As shown in Table 2, the results of *C. parvum* antigen detection among 820 fecal samples collected from different farm animals showed 268 out of 820 samples were positive recording an infection rate of 32.7% of animals including 49% (50/102) in buffalo calves, 57.5% (69/120) of cattle calves, 23% (23/100) of lambs, 25.5% (25/98) in goat kids, 35% (28/80) in buffaloes, 43.3% (26/60) in cattle, 20.6% (33/160) in sheep, and 14% (14/100) in goats. Using PCR to evaluate the positivity of the sandwich ELISA results, the total PCR positive samples were 263 out of in-house sandwich ELISA positive samples 268 (98.13%). The PCR positive samples were 49/50 (98%), 69/69 (100%), 22/23 (95.65%), 24/25 (96%), 27/28 (96.43%), 26/26 (100%), 32/33 (96.97%) and 14/14 (100%) in buffalo calves, cattle calves, lambs, goat kids, adult buffaloes, adult cattle, sheep and goats’ fecal samples, respectively.

**Table 2:** Comparison between conventional MZN, in-house sandwich ELISA and commercial sandwich ELISA kit for diagnosis of cryptosporidiosis in farm animal species

Animal species	Diagnostic test			
	No. of fecal samples	MZN positive (%)	In-house sandwich LISA Positive (%)	Commercial ELISA kit positive (%)
Buffalo calves	102	46/102 (45.1%)	50/102 (49%)	47/102 (46.1%)
Cattle calves	120	60/120 (50%)	69/120 (57.5%)	64/120 (53.3%)
Lambs	100	20/100 (20%)	23/100 (23%)	21/100 (21%)
Goat kids	98	18/98 (18.4%)	25/98 (25.5%)	19/98 (19.4%)
Adult buffaloes	80	25/80 (31.25%)	28/80 (35%)	26/80 (32.5%)
Adult cattle	60	23/60 (38.3%)	26/60 (43.3%)	24/60 (40%)
Sheep	160	30/160 (18.8%)	33/160 (20.6%)	31/160 (19.4%)
Goats	100	11/100 (11%)	14/100 (14%)	13/100 (13%)
Total	820	233/820 (28.4%)	268/820 (32.7%)	245/820 (29.9%)
χ^2^		50.5*	48.65*	33.36*

*P < 0.001: Statistically significant

The presence of *C. parvum* antigen in animals’ fecal samples (*n* = 820) of the different hosts was confirmed by a commercial sandwich ELISA kit as the commercially available method. The total infection rate was 29.9% (245/820), where it was 46.1% (47/102), 53.3% (64/120), 21% (21/100), 19.4% (19/98), 32.5% (26/80), 40% (24/60), 19.4% (31/160) and 13% (13/100) in buffalo calves, cattle calves, lambs, goat kids, adult buffaloes, adult cattle, sheep and goats’ fecal samples, respectively (Table 2).

## Discussion

Livestock offers financial possibilities, income to farming communities, draught power and investment opportunities (Ebiyo and Haile 2022) and infectious diseases are a great challenge for this industry. *Cryptosporidium* spp. are widely prevalent apicomplexan protozoans which had been identified as significant contributors to calf and small ruminant diarrhea worldwide (Cho and Yoon 2014; Mahmoudi et al. 2021). Molecular characterization for *Cryptosporidium* spp. had validated 30 species that infect mammals, birds, amphibians, reptiles and fish (Fayer et al. 2010) with more additional species and genotypes identified every year (Šlapeta 2013) where, *C. parvum* was found to be the most dominant species in farm animals (Tomazic et al. 2018), besides being the most zoonotic species (Feng et al. 2018; Cunha et al. 2019). In our study, we detected *C. parvum* in the buffalo calves from Giza (GenBank: OQ121955.1), cattle calves from Cairo (GenBank: OR029973.1) and adult buffaloes from Beni-suef (GenBank: PP316107.1) and these isolates shared 100% homology with other Egyptian *C. parvum* isolates submitted to the GenBank using COWP gene (GenBank: ON730707.1, ON730708.1, ON351679.1 and OM817461.1) from buffaloes in Beni-suef, buffaloes in Giza, camels in Northwestern coastal zone and sand rats in North Coast, respectively). This presence of *C. parvum* in young and adult age classes could imply critical animal and public health concerns (Díaz et al. 2021). Sheep and goats are common hosts of *C. parvum*, but they can also be infected with *C. xiaoi*, C. *hominis*, *C. andersoni*,* C. ubiquitum* and many other *Cryptosporidium* species (Fiuza et al. 2011; Zhang et al. 2020). In the present study, we identified *C. hominis* in sheep from Cairo (GenBank: OR029972.1) which shared 100% homology with (GenBank: KX365892.1, MK033082.1 and OR029971.1) isolated from humans in Cairo, Egypt. In parallel, other studies detected *C. hominis*, the major human infective *Cryptosporidium* species, occasionally in sheep (Pritchard et al. 2008; Kaupke et al. 2017; Lang et al. 2023), cattle and other animals (Feng et al. 2018). We also detected *C. andersoni* in goats from Giza (GenBank: PP316108.1) which was identical (100%) to (GenBank: AB514044.1 and AB514043.1) detected in cattle calves from Kafr El Sheikh, Egypt and (GenBank: KF747672.1 and DQ989571.1) in camels from China. In related studies, *C. andersoni* was also detected in goats in different locations of China (Zhong et al. 2018; Lang et al. 2023).

Diagnosis of cryptosporidiosis is done either microscopically, immunologically or by molecular methods. In our study, we detected *Cryptosporidium* oocysts in 233/820 fecal samples (28.4%) among the selected governorates using conventional microscopy. Moreover, the percentage of *Cryptosporidium* infection was more dominant in young animals (buffalo calves, cattle calves, lambs and goat kids) compared to adults (adult buffaloes, cattle, sheep and goats). This coincides with Joute et al. (2016), Elmahallawy et al. (2022) and Chen et al. (2022). In addition, our results agreed with Santín (2020) who stated that cryptosporidiosis is more dominant in neonatal ruminants. Cai et al. (2019) found that *Cryptosporidium* prevalence in cattle calves aged ≤ 12 months was significantly higher (22.5%) than others aged > 12 months (9.5%). Furthermore, Paraud and Chartier (2012) reported that cryptosporidiosis in neonate small ruminants varied according to species due to differences in immune response against *Cryptosporidium* among host species. It was assumed that the age-related decrease in the infection rates of *Cryptosporidium* spp. reflects the establishment of immunity by previous infection (Guy et al. 2022). Also, variation in the infection rates could be due to sampling size, rearing system, season of sample collection, hygienic measures, environmental and managemental factors (Mahfouz et al. 2014).

Detection of *Cryptosporidium* specific antigen in fecal samples is likely an appropriate test for its diagnosis (Gerace et al. 2019) especially in large farms. In our study, the purified anti-*C. parvum* antigen IgG PAbs was characterized by two bands the heavy chains 50 KDa and the light chain 30 KDa of immunoglobulin using 10% SDS-PAGE. This result was somewhat similar to the two bands 53 and 31 KDa (Heavy and light chains) of the purified *C. parvum* oocyst IgG PAbs using 12.5% SDS-PAGE to be used in diagnosis of cryptosporidiosis in stool and serum samples of infected calves (Farid et al. 2023). This slight difference could be attributed to the crude antigen used, the purification procedure, and the SDS-PAGE slab gel percentage used for characterization. In our study, the purified anti‑*C. parvum* IgG PAbs had high diagnostic potential and strong binding activities that succeeded in monitoring the oocysts shed in fecal samples collected from mice experimentally infected with *C. parvum* as proved by our in-house sandwich ELISA based on two capture antibodies.

In-house sandwich ELISA involved in this study also achieved sensitivity and specificity with validation and agreement higher than those of the MZN staining and commercial sandwich ELISA kit. Our results were higher than the sensitivity (63.6% and 40.9%) and specificity (75.9% and 78.9%) of enzyme immune assay (EIA), and ELISA, respectively (Danišová et al. 2018). Higher results were shown by Maher et al. (2022) who detected a specificity of 92.72% and a sensitivity of 97% when used a sandwich ELISA with PAbs in fecal samples. In another study, Abdou et al. (2022) found that immunochromatographic assays (IC) had the highest sensitivity (74.07%) while MZN had the highest specificity (98.29%) in the detection of *Cryptosporidium* in cattle feces. These differences in sensitivity and specificity might be attributed to the finding that not all commercially produced antibodies can recognize all *Cryptosporidium* spp. oocysts’ antigens, although EIA tests detect all soluble and insoluble antigens (Danišová et al. 2018). Also, antibodies in the commercial kits weakly respond to the antigens of some species which are genetically distant from species *C. parvum* and *C. hominis*, which serve as a basis to producing these tests (Smith et al. 2008). Khurana and Chaudhary (2018) stated that antigen screening techniques are more recommended due to their higher sensitivity and ability to monitor acute infection.

In our study, the purification process of the prepared IgG polyclonal antibodies (IgG PAbs) increased the sensitivity and specificity of the immunodiagnostic tests for diagnosis of parasitic infection, and this came in agreement with Toaleb and Abdel-Rahman (2021)Aboelsoued et al. 2022a; Darwish et al. (2024); Toaleb and Shaapan (2024). The detection of *C. parvum* oocyst antigen in 820 fecal samples from different species of farm animals using in-house sandwich ELISA based on the purified anti-*C. parvum* oocyst IgG PAbs showed a higher infection rate (32.7%) than the commercial ELISA kit (29.9%) and conventional MZN (28.4%) results. Khurana et al. (2012) and Sharma and Busang (2015) reported that ELISA was found to be more sensitive than MZN. In contrast, no statistical difference between ELISA and MZN in the detection of *Cryptosporidium* was recorded by Al-Megrin (2015) Moreover, although microscopical examination is still commonly performed as it’s cost efficient and simple, it lacks sensitivity (McHardy et al. 2014) and its accuracy depends mainly on expert technicians (Ahmed and Karanis 2018) as sometimes the stained smears couldn’t discriminate between *Cryptosporidium* oocysts and other spherical objects of the same size (such as yeast) (Connelly et al. 2013). In addition, Khurana et al. (2012) found that many samples could be tested quickly by sandwich ELISA with a high specificity range of 98–100%. Also, Khurana and Chaudhary (2018) reported that fresh, formalin-preserved, or frozen samples could be successfully tested by this assay. These multiple and quick tests in a single response device give the sandwich ELISA the superiority than fecal microscopic examination (Farid et al. 2023). Interestingly, after running a PCR for the in-house sandwich ELISA positive samples, we found that the infection rate was 98.13% (263/268) confirming results of our in-house sandwich ELISA. This slight difference might be due to insufficient oocysts’ concentration or failure of oocyst disruption in samples subjected to DNA extraction. This coincides with Elmahallawy et al. (2022) who found 2 samples that were positive by microscopy and were negative by PCR. Fecal constituents such as complex polysaccharides, bile salts and bilirubin could inhibit PCR even being at low concentrations (Monteiro et al. 1997).

Our in-house sandwich ELISA, based on prepared anti-*C. parvum* IgG PAbs whole molecules, is a rapid and easy assay, with high sensitivity (98%) and specificity (100%) for the early diagnosis of *Cryptosporidium* infection in feces of small ruminants, buffaloes, and cattle. Antigens detection rather than antibodies is considered a more reliable method to evaluate the status of infection and monitor the efficacy of treatment. In addition, it could offer significant medical and veterinary importance, helping in early detection of cryptosporidiosis. The molecular analysis in different animals determines species allocation and the disease’s national impact.

## Data Availability

All data generated and analyzed in this study are included in the published manuscript. The nucleotide sequences of *C. parvum*, *C. andersoni* and *C. hominis* for the COWP gene obtained in this study have been submitted to the GenBank, GenBank accession numbers: OQ121955.1, OR029973.1, PP316107.1, PP316108.1, OR029972.1, (https://www.ncbi.nlm.nih.gov/genbank).
